# Testing Scalability of a Diabetes Self-Management Intervention in Northern Mexico: An Ecological Approach

**DOI:** 10.3389/fpubh.2021.617468

**Published:** 2021-08-18

**Authors:** Benjamin Aceves, Catalina A. Denman, Maia Ingram, Jose Francisco Torres, Tomas Nuño, David O. Garcia, Purnima Madhivanan, Cecilia B. Rosales

**Affiliations:** ^1^Public Health Practice and Translational Research, University of Arizona, Tucson, AZ, United States; ^2^El Colegio de Sonora, Hermosillo, Mexico; ^3^Sonoran Ministry of Health, San Luis Rio Colorado, Mexico

**Keywords:** chronic disease, diabetes, mexico, self-management, implementation

## Abstract

**Background:** Type 2 diabetes mellitus (T2DM) has become a major issue in Mexico, reporting almost 100,000 attributable deaths in 2016. Low-income Mexican citizens who face various issues associated with T2DM, including the lack of access to self-management services, are particularly affected by the condition. Health centers have been designated to serve T2DM patients by providing resources on chronic disease prevention. *Meta Salud Diabetes* (MSD) is a self-management intervention developed to address cardiovascular complications and other health issues within the T2DM population, which have been proven effective and useful for health centers. The intervention was designed for T2DM support groups—*grupos de ayuda mutua* (GAMs) located within health centers.

**Methods:** From February to June 2019, a binational research team conducted a test scale-up study in Northwest Sonora under the Ministry of Health utilizing the Institute for Healthcare Improvement Framework for scaling up health interventions. Investigators worked in collaboration and trained 19 stakeholders from a regional health system identified from various ecological levels on MSD and implementation process.

**Results:** All five GAMs within the regional health system received and completed the intervention. In total, 72 participants were enrolled with behavioral and biological [HbA1c, blood pressure, body mass index (BMI)] measures taken at baseline. Post-intervention measurements were taken from 72% of participants who completed the intervention. Statistical analysis demonstrated improved behavioral and biological measures when comparing baseline to post-intervention, specifically statistically significant improvements in HbA1c and sugar-sweetened beverage consumption. Implementation fidelity (IF) measures indicated extensive adherence to the intervention curriculum, and moderators specifically demonstrated influences on implementation. Stakeholders from various ecological levels provided support to those facilitating the MSD intervention by allotting time and resources to properly prepare for sessions. An implementation coordinator from the regional health office assisted MSD facilitators by resolving barriers to implementation and worked toward federal accreditation for GAMs to receive additional funding.

**Conclusion:** Results provide evidence for using regional health systems as a scalable unit when implementing chronic disease self-management interventions state- and nationwide. This study will help inform future efforts to scale up the health intervention in various states throughout Mexico.

**Clinical Trial Registration:**www.ClinicalTrials.gov; https://www.clinicaltrials.gov/ct2/show/NCT02804698?term=NCT02804698&draw=2&rank=1, identifier: NCT02804698.

## Background

In 2016, the Mexican Ministry of Health for the first time in its history announced an epidemiologic emergency due to type 2 diabetes mellitus (T2DM), affirming the disease was associated with almost 100,000 attributable deaths per year (1). In addition to the high prevalence of T2DM, people living with the chronic disease are at a higher risk for cardiovascular diseases (CVDs), which is the leading cause of death in Mexico (2). Generally, non-communicable diseases (NCDs) were related to ~80% of deaths in Mexico for 2015, which is significantly greater than the 71% seen globally the same year (3). The high prevalence of NCD risk factors nationwide is linked to the high prevalence of obesity (40.2% among women and 30.5% among men) and hypertension (20.9% among women and 15.3% among men) among adults over 20 years of age (4, 5). These risk factors are influenced by a host of social, economic, and environmental contributors, such as a lack of healthy social networks and support, living in food swamps, and issues with community safety due to violence in the country (6–9). All contributors disproportionately affect aging low-income populations in Mexico, who in addition have limited access to quality health care increasing their risk for NCDs and associated complications (6, 9).

Support groups—*grupos de ayuda mutua* (GAMs)—for people with NCDs are part of a comprehensive strategy under the federal and state Ministries of Health to provide health care to this vulnerable population (10). The GAMs were conceptualized as a space for individuals to access more social support and resources to healthy lifestyle change and maintenance (10). People are first diagnosed as having hypertension or T2DM by their provider, then referred to a GAM at a local health center (10). This ensures that participants are simultaneously receiving social, educational support, and clinical treatment (10). The groups demonstrate part of the efforts made by the Ministry of Health to help curb the increasingly alarmingly epidemic. GAMs have demonstrated some success in supporting patient self-management by connecting them to educational resources and social support (11). However, as stated in Mexico's Specific Action Program on Diabetes Mellitus 2007–2012, a need continues to exist for the implementation of a scalable, evidence-based program that addresses healthy lifestyle promotion and self-management for T2DM patients (12). A US–Mexico binational effort was undertaken in the state of Sonora between two academic institutions in collaboration with the state public health system to fill this void through the development and implementation of the *Meta Salud Diabetes (MSD)* intervention.

### History of the MSD Intervention

*Meta Salud Diabetes* is the result of ongoing efforts to address NCD self-management and prevention in the US–Mexico border region through binational collaborative research. *Su Corazon Su Vida* was the initial effort, a lifestyle intervention that sought to decrease risk factors associated with CVD, which was then collaboratively adapted to produce *Pasos Adelante* for a US population located along the Arizona–Sonora border region (13). This intervention was later adapted for a Mexican population, resulting in *Meta Salud*, a community-based, primary prevention intervention that aimed to prevent obesity and other NCD through addressing behavioral and biological risk factors (14). The success of *Meta Salud* at the community level led to the development of *MSD*, which was designed to reduce CVD complications among a patient population with diabetes.

The MSD intervention consists of 13 evidence-based weekly sessions designed for patients living with T2DM and attending the GAMs. The participatory focused curriculum centers around culturally competent group-based interactive activities (15). Each session is facilitated by trained health center staff and addresses risk behaviors for CVD, specifically helping participants identify strategies to improving health outcomes through didactic learning, social support, and role modeling (15). Intervention effectiveness was assessed utilizing a cluster-randomized design by measuring changes in behavioral and clinical risk factors among participants at health centers (*n* = 22) across the state of Sonora (15). In addition, an implementation study identified strategies, facilitators, and barriers to adopting and integrating the intervention into the Ministry of Health provider system in the state (15, 16). Results from analyses of stakeholder focus groups and observational data revealed contextual factors (i.e., staff training, institutional support, material resources) for consideration to ensure full potential of the intervention (16). Overall, the implementation study indicated positive reception of *MSD* within the public health system, and concurrently, it has produced rich qualitative data that indicate a need to scale up the intervention into the health systems given high acceptance and urgent need (16). These findings, in conjunction with productive relationships and buy-in from government personnel, created an opportunity to develop and test a scalable unit as the first step in broader scale-up across Mexican state health systems. The investigators identified regional health systems in Sonora, or as termed in Mexico jurisdictions, as a unit of analysis to study the process. Jurisdiction VI, the northwest region of the state of Sonora bordering the United States, volunteered to be the site for developing and testing a scalable unit. In developing and testing a regional health system as a scalable unit, the investigators hope to contribute to the evidence surrounding these strategies and pathways.

## Methods

Given the plethora of pathways for scale-up developed in recent years, it is essential to select a scale-up strategy that is evidence-based and scientifically rigorous, but also suitable for the needs of partners and trajectory of the program or intervention (17). The Institute for Healthcare Improvement (IHI) Framework for Going to Full Scale was utilized to assess the MSD intervention within the trajectory of scale-up (18). The IHI Framework has been utilized in low- and middle-income countries (LMIC) countries to scale up interventions and programs across health systems nationwide (18). The framework insists that scale-up should be understood within four chronological stages: set-up, develop the scalable unit, test of scale-up, and go to full scale (18). Acknowledging the original testing of the intervention within health centers and the continued partnership with the Sonoran Ministry of Health as the set-up stage, the research team identified the need to develop the scalable unit (15, 18).

The IHI Framework identifies this stage as a crucial time to communicate the importance of the intervention to leadership, establish the infrastructure, and create learning systems to vet innovative strategies through testing (18). Given the need for communication, infrastructure, and innovation, the research team developed a scale-up approach grounded in the Social Ecological Model (SEM) to test within Jurisdiction VI (19). Addressing multiple ecological levels was central to scaling up *MSD* within the regional health system among an array of stakeholders (20). The SEM has been found to be a productive model for framing roles of stakeholders within a CVD prevention intervention in the US–Mexico border, specifically when engaging government institutions within the process (21). The model provides a context to understanding and dissecting dynamic social–professional relationships that exist between stakeholders at various levels—intrapersonal (patients), organizational (facilitators, health center directors), and policy level (regional office staff, Jurisdiction VI director) (22).

Additionally, the social–ecological approach to understanding the power dynamics at work within existing social systems has been extensively used within implementation science and among health researchers across the globe (23, 24). Given the emphasis of the SEM on engaging stakeholders from different ecological levels, the investigators established a scale-up approach in collaboration with the health centers, jurisdictional leadership, and the Ministry of Health (19, 20). The strategies to the approach included create a continuous stream of communication between the different GAMs and the regional office, designate a coordinator to identify needs and advocate for infrastructure for the intervention, and consistently learn and adapt from the scale-up process throughout its implementation.

**Figure 1:**
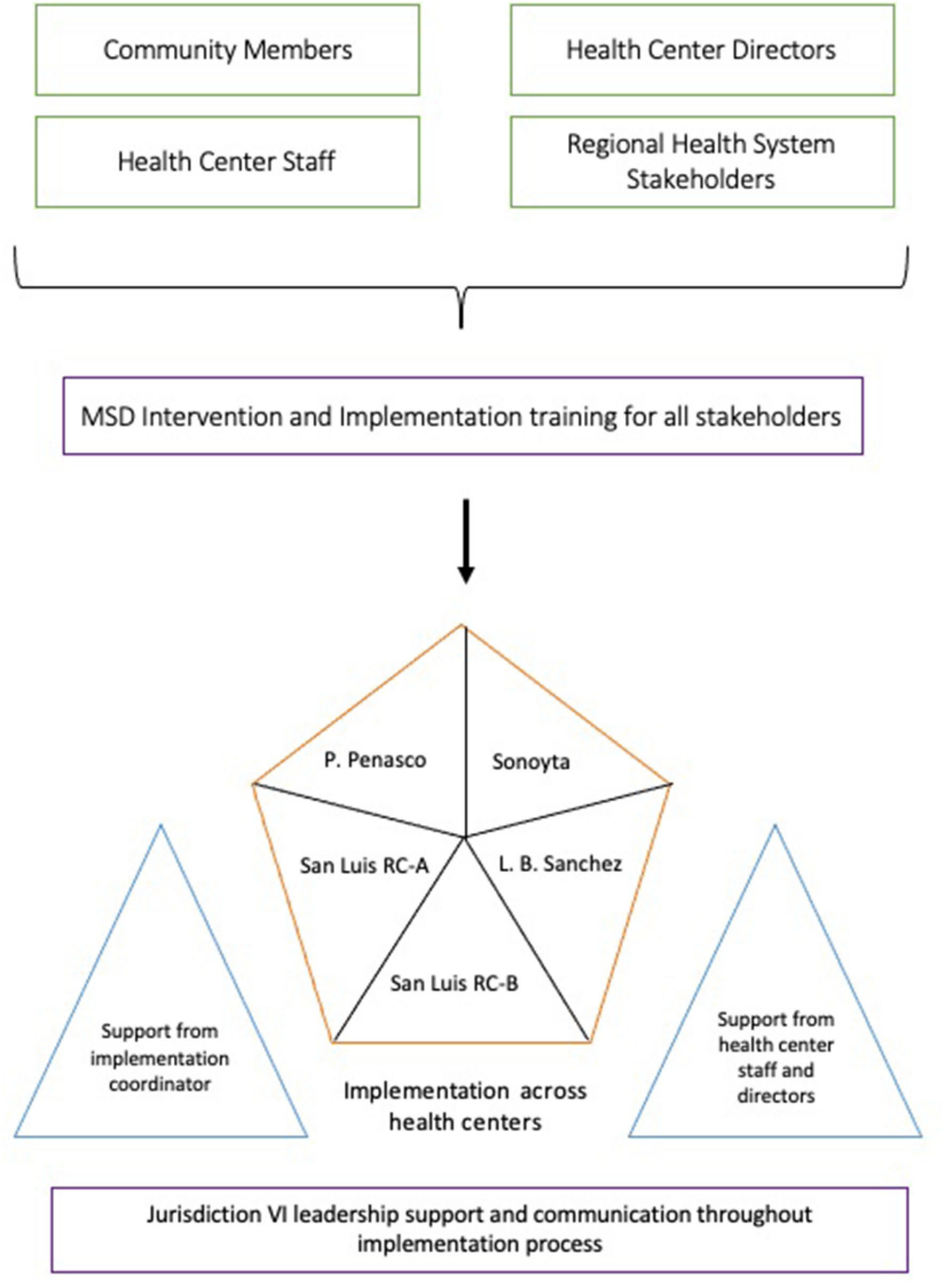
Ecological scale-up approach.

As seen in Figure 1, the ecological approach to scale-up involved creating foundational infrastructure by training various stakeholders from distinct levels on the *MSD* intervention and the implementation process during scale-up. Jurisdictional, health center, GAM leadership, and community members were invited to provide varying perspectives and feedback during the training. The 6-h one-day MSD training addressed the following topics within the context of implementation:

- T2DM and CVD risk factors.
- Education on the role of support groups in improving disease management, promoting sustainable change, preventing complications, and assisting to address barriers and access to care.
- Lifestyle changes and education related to nutrition and physical activity that promote secondary prevention.
- Interactive learning strategies.
- Building self-efficacy for facilitators.
- Potential implementation facilitators (IF) and barriers.

All five GAMs within four health centers in the jurisdiction were included. A designated implementation coordinator supported facilitators with sessions during the implementation of the MSD intervention at all sites. This coordinator was also responsible for managing communication between the regional office and each GAM at health centers, as well as advocating for any necessary resources needed by facilitators. Additionally, facilitators received direct support from the health center staff such as other nurses, doctors, and directors.

Investigators used biological and behavior measures associated with CVD risk to ensure beneficence for participants and comprehensive IF measures to understand and assess the scale-up process across the entire system through the implementation process. Specifically, the Carroll et al. framework for IF was utilized because of the comprehensive approach that assisted investigators in assessing scale-up across the health system (25). The following IF adherence measures and adapted moderators were assessed: coverage, duration, frequency, content, intervention complexity, strategies to facilitating implementation, quality of delivery, and participant responsiveness (25).

### Eligibility, Demographics, Biological, and Behavioral Measures

Two Spanish-speaking trained clinical researchers collected baseline demographics and biological measures prior to the first session and following informed consent by every participant. Participants were required to be a diagnosed with T2DM from their provider. Demographic questions included gender, income level, age, health coverage, and length of membership within the GAM. The biological measures taken at baseline and post-intervention included glycated hemoglobin (HbA1c), blood pressure, and body mass index (BMI) calculated from height and weight measurements. In addition, two questions measured sugar-sweetened beverage consumption and sedentary time at both time points. Participant consent and data collection was conducted under protocol approved by the University of Arizona Institutional IRB.

### Statistical Analysis

Data analysis was conducted using Stata version 16.0 (StataCorp, College Station, Texas). Proportions were calculated for categorical data, and means and standard deviations were calculated for continuous data. Descriptive statistics were calculated for baseline demographic variables. To compare baseline to post-intervention mean differences, the paired *t*-test was used with the pre-/post-measures taken from participants that completed the intervention. The paired *t*-test and chi-square test of association were conducted to assess the baseline differences between participants that completed the intervention with those who dropped.

### Implementation Fidelity

Coverage, duration, frequency, and content were measured in order to assess adherence to the MSD intervention (25). All measures were evaluated at an aggregate level in order to assess IF within the entire regional health system of the Jurisdiction VI. As seen in Table 1, coverage and frequency were measured using facilitator-reported completion rates among all participants and overall attendance. Completion rate was defined as participants who attended a minimum of 11 sessions, including the first and final session. Duration and content were measured using ~30% of randomly selected sessions (2, 4, 8, 12). Facilitators self-reported start and end time points for each selected session, and the mean time was calculated across all groups for each session. Content was measured using a self-reported content checklist. For each randomly selected session, facilitators checked if the GAM completed overarching activities. For each activity completed, they received a score of 1 or 0 for non-completion; in total, each session has six overarching activities. The totals were used to calculate a content score.

**Table 1:** Implementation fidelity measures and assessments.

**IF component**	**Measures and assessments**
**Adherence**	
Coverage and Frequency	• Completion rate of intervention
Duration	• Randomly selected sessions - mean time
Content	• Content scores- calculated from checklists on the six overarching activities completed
**Moderators**	
Intervention complexity	• Baseline/post-training assessment of Jurisdiction VI stakeholders •Field notes and session observations
Strategies to facilitate implementation	• Baseline/post-training assessment of Jurisdiction VI stakeholders •Field notes and session observations
Quality of delivery	• Baseline/post-training assessment of Jurisdiction VI stakeholders •Field notes and session observations
Participant responsiveness	• Field notes and session observations •Post-intervention open-ended question

Various methods were used to assess moderators to IF. Intervention complexity, strategies to facilitating implementation, and quality of delivery were initially assessed and addressed through the training for Jurisdiction VI stakeholders (25). During the training, stakeholders were given baseline/post-training assessments to gauge knowledge on the intervention and corresponding theoretical frameworks, as well as their self-efficacy on their ability to facilitate and implement MSD. Session observations conducted during sessions 1, 7, 8, and 13 by the principal investigator (BA) and field notes taken throughout the intervention were used to assess all moderators. Field notes and session observations have been proven critical components to assessing implementation processes and dynamics in previous studies (26–28). Participant responsiveness was additionally assessed through an open-ended exit question inquiring about their overall experience with the intervention with additional probing conducted by the interviewer for short responses. All participant responses, field notes, and session observations were analyzed using direct content analysis with support by NVivo 12 software (29, 30). In addition, Standards for Reporting Implementation Studies: the StaRI checklist for completion was used a guideline to transparently report on the study in its entirety (31). Qualitative data on feedback of patient participants surrounding their experience, benefits with MSD, along with opportunities from improvement, facilitators, and barriers have been previously published (16, 32). In this manuscript, we focus more generally on reporting the moderating factors that may impact scaling an intervention within an LMIC health system.

## Results

**Figure 2:**
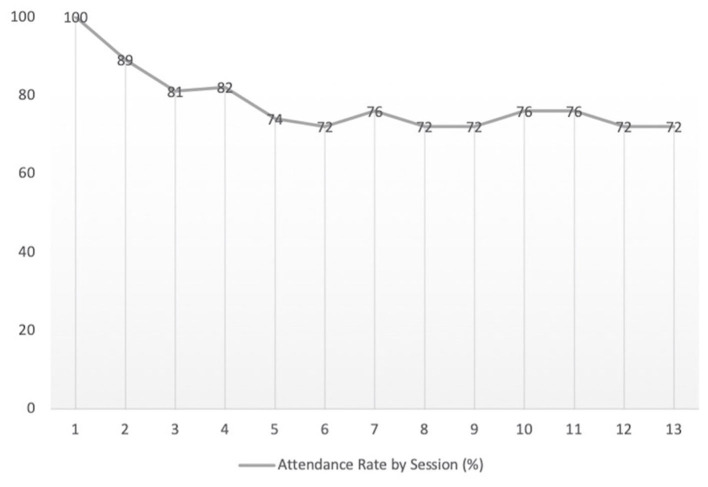
Overall intervention attendance (*n* = 72).

A total of 72 participants enrolled in the study in all five groups located within the four health centers. The majority of participants identified as female (74%), and the average age was 59 years (SD: ±11). Over 50% of the monthly income of participants was ≥ US$206, while 25% of participants were below the Mexican poverty monthly income threshold of US$102. Approximately 51% of participants had been a member of the GAM for over a year or more, and 87.5% were covered by *Seguro Popular* program. As seen in Figure 2, a total of 52 participants (72%) completed the intervention.

As seen in Table 2, HbA1c decreased significantly from 7.12% (*SD* = 1.78) at baseline to 6.49% (*SD* = 1.30) post-intervention among participants who completed the 13 weeks (*p* = 0.039). Sugar-sweetened beverage consumption also decreased from 6.71 cups (*SD* = 7.95) to 3.76 cups (*SD* = 6.11) per week (*p* = 0.038). Additionally, there was a decrease in BMI, systolic blood pressure, diastolic blood pressure, and average sedentary time; however, these changes were not statistically significant.

**Table 2:** Behavioral and biological outcomes for participants (*n* = 52).

**Measure**	**Baseline** **mean**	**Post-Intervention** **mean**	**Difference** **(standard error)**	***p*-value**
HbA1c (%)	7.12 ± 1.78	6.49 ± 1.30	0.63 (0.30)	0.039^*^
BMI (kg/m^2^)	31.89 ± 5.09	31.73 ± 4.99	0.16 (0.98)	0.869
Systolic blood pressure (mmHg)	139.59 ± 3.10	133.5 ± 2.48	6.17 (3.97)	0.123
Diastolic blood pressure (mmHg)	82.55 ± 1.55	81.04 ± 7.63	1.52 (1.88)	0.421
Average sedentary time (total minutes in a day)	239.8 ± 191.18	222.69 ± 170.05	17.10 (35.79)	0.634
Sugar-sweetened beverage consumption (# of 250 ml cups per week)	6.71 ± 7.95	3.76 ± 6.11	2.94 (1.4)	0.038^*^

* *Statistically significant values*.

### Training Among Jurisdiction VI Stakeholders

A total of 19 individuals from Jurisdiction VI attended the MSD implementation training. These stakeholders identified with the following professions or roles within the health system: eight as nurses; three as doctors; four as community members/did not identify; and one each of the following dietitian, psychologist, physical activity coordinator, and health administrator. They represented various stakeholders at various ecological levels involved with implementing the MSD within the regional health system.

As seen in Table 3, stakeholders demonstrated increased knowledge surrounding the intervention structure and curriculum with a mean change of 1.53 (*SE* = 0.34) when comparing baseline to post-training assessment. Additionally, an increased understanding of theories and framework that format GAMs was observed with a mean change of 1.37 (*SE* = 0.35) among the trained stakeholders. Self-efficacy also increased among stakeholders after the training, specifically they felt better able to facilitate the *MSD* sessions in an interactive and participatory manner with a mean change of 1.10 (*SE* = 0.31) from baseline. Self-efficacy of stakeholders to addressing barriers and facilitators to implementing *MSD* also increased with a mean change of 1.22 (*SE* = 1.22).

**Table 3:** Mean Scale Outcome at Baseline and Mean Changes after Meta Salud Diabetes Training for Stakeholders (*n* = 19).

**Training** **objective**	**Baseline** **(SD)**	**Mean change (SE)**	**95% confidence** **intervals**	***p*-value**
**Knowledge**				
MSD intervention structure and curriculum	2.53 (1.12)	1.53 (0.34)	(0.84, 2.21)	0.000
Theories and frameworks used to format GAMs	2.68 (1.06)	1.37 (0.35)	(0.66, 2.07)	0.000
**Self-efficacy**				
Facilitate the MSD sessions in an interactive and participatory manner	3.37 (1.21)	1.10 (0.31)	(0.47, 1.73)	0.001
Address barriers and facilitators to implementing MSD	3 (1.29)	1.22 (0.37)	(0.48, 1.96)	0.002

### Session Duration and Content Coverage Scores

As seen in Table 4, the overall mean time of randomly selected sessions was 162.8 min (*SD* = 48.73). The large values of standard deviation indicate the variation between the time durations taken to conduct each session by the groups. Overall, the content score (measurement used to indicate the completion of the intervention curriculum) across groups was 5.35 (*SD* = 0.67) with maximum possible being 6.0. The lowest overall mean content score across sessions was 5 (*SD* = 1.0) for session 8 and the highest 5.6 (*SD* = 0.55) for session 12. As seen in Table 1, the session with the lowest duration time also contained the lowest coverage score. Conversely, the session with the highest duration time contained the coverage scores closest to the maximum score of 6. These values indicate that sessions with longer mean durations had higher content scores and therefore increased exposure to intervention for participants. Mean duration times indicated that the intervention sessions took significantly longer [between 35.5 and 54.8 min] than the prescribed 120-min time in the intervention curriculum.

**Table 4:** Duration and content for each session of meta salud diabetes (*n* = 5).

**Session** **number**	**Mean duration** **minutes**	**Mean content scores** **range 0–6**
2	155.2 (52.95)	5.4 (0.55)
4	161.6 (32.75)	5.4 (0.55)
8	159.6 (60.95)	5 (1)
12	174.8 (58.5)	5.6 (0.55)
Overall	162.8 (48.73)	5.35 (0.67)

*( ) – Standard Deviation for mean value*.

### Assessing and Addressing Moderators

Intervention complexity, strategies to facilitate implementation, and quality of delivery were initially addressed primarily through the training on intervention structure/objectives and interactive role-play scenarios. Field notes and session observations indicated that the implementation coordinator assisted stakeholders by: addressing questions; clarifying any confusion surrounding the intervention topics; and problem-solving barriers to implementing sessions. Two consistent barriers to implementation were securing space and resources for conducting each session. The implementation coordinator indicated in one instance,

“*The nurse and leaders of the GAMs in the health center need help in running the session so I step in, but the health centers also support by providing additional resources such as office supplies, drinking water for participants, and measuring weight or blood pressure for participants.”*

Given the health center directors and investment of regional director in the intervention, the majority of the health center staff and stakeholders from the Jurisdiction VI office actively engaged in implementing the intervention.

However, the issue of space tended to be more difficult to address given competing programs and health services within the health center. One health center consistently excluded physical activities during sessions because it was not possible to conduct with the limited space available. In addition, the implementation coordinator was motivated to assist with the sessions in order to ensure consistency to gain federal accreditation of three GAMs in the jurisdiction from the federal government. One additional barrier to implementation of the intervention was the facilitators feeling overwhelmed by the responsibilities of leading each session. Lastly, the implementation coordinator was central to facilitating implementation by establishing communication between stakeholders and the regional offices, which contributed to the deconstruction of the traditional hierarchical chain of commander system for programs. The coordinator was able to communicate the needs of stakeholders at the local level to the central regional office, and in turn resources research GAMs more quickly. Overall, the implementation coordinator was motivated to facilitate implementation in order to gain federal accreditation for the GAMs in Jurisdiction VI.

In terms of quality of delivery, most stakeholders were able to actively engage participants with the intervention curriculum and obtain additional assistance from other health personnel at centers on various health topics related to T2DM. All health centers directors were present in at least one of the sessions, and field notes indicated that health directors provided support in terms of protected time and resources for the groups, such as paper supplies, glucometers with test strips, and drinking water for session. Feedback was provided after session observations to stakeholders to enhance quality of delivery. In general, stakeholders appreciated the independence and protected time dedicated to preparing for the intervention, which they believe helped improve the quality of delivery and motivated them to lead the sessions in an interactive manner. In addition, strong social relationships of stakeholders tended to result in increased participation and self-efficacy as observed by investigators during sessions. The social cohesion and connectedness contributed to participants delving into more personal topics that reflected their lived experiences of people living with T2DM, particularly related to lacking resources or support.

Based on session observations and field notes, responsiveness of participants was reported as interactive and engaged during the intervention. Additionally, responses from exit questions indicated that participants felt they gained more energy to engage in healthy behaviors, increased social interaction to share experiences, and knowledge surrounding their health status. In terms of gained energy, one participant stated, “*It has been good because I have been able to be more active and do more exercise*.” Other participants also indicated that they had more energy to engage in daily activities related to domestic and professional work. Participants additionally enjoyed the increased social interactions and knowledge surrounding their health status, as stated by one participant, “*I have learned a lot about diabetes and how to live with the disease, and I learned that I was misinformed and now also I have been able to interact with others about my experiences*.” Similarly, another participant mentioned more interaction with health professionals, “*I have felt great! I really like it I haven't missed a session. I like the lessons about diabetes, it is a beautiful program that teaches us about ourselves and connects us to the nurse*.” Overall, participants expressed positive responses when explaining their experience with *MSD* and felt it facilitated lifestyle change.

## Discussion

Mexico is currently facing an epidemic related to NCDs, and every level of government in the country must continue to explore innovative solutions. Evidence-based programs and interventions are part of the answer; however, research is needed to build knowledge around strategies and pathways for implementation (17). In developing and testing a regional health system as a scalable unit, the investigators hope to contribute to the evidence surrounding these strategies and pathways. The biological markers collected provide evidence on the sustained intervention benefits to participants during scale-up. While the adherence measures indicate a consistent patient involvement and high IF to the intervention, there still exists a need to provide adaptability strategies for health centers facing time constraints. The moderating factors impacting scaling demonstrated the central role of the implementation coordinator, a need for adaptation strategies, importance of stakeholder motivation and social networks, and utility of strong communication and learning systems.

The implementation coordinator facilitated communication between levels to ensure that implementation was achieved—in effect increasing collaboration between health staff, resources for facilitators, and support throughout the jurisdiction for the GAMs and intervention. As a result, it deconstructed some of the rigid top-down power structures that exist within a Mexican regional health system through connecting facilitators directly to the regional health office. This approach and the IF assessment also aided researchers and jurisdiction leadership in understanding consistent barriers faced by centers, motivations of various stakeholders for scaling the intervention, and the overall structures needed for learning systems. As noted in assessing several of the moderators, the implementation coordinator assisted in problem solving such as helping in facilitating sessions, but also advocating for resources that were readily available, and more easily facilitated sessions.

The implementation coordinator was also an important component of understanding adaptability of the intervention. He was able to work with health centers to protect fidelity of the intervention, but also ensured that the centers had the capacity and the resources to conduct each session. This translated to adapting the intervention at times, such stepping in as the health expert for the session, modifying activities for abilities and available resources of participants, or working to find adequate spaces.

The adaptation strategies mentioned above also helped to identify consistent barriers faced by health centers during scale-up such as finding adequate stakeholders to conduct the intervention. Competing programs were constantly being introduced or continued, particularly with larger health centers, which resulted in time constraints to conduct each session. This added to a limited ability of stakeholders to adapt and prepare rooms according to the session activities. This identified a need to build-in flexible activities according to the space and time allotted for sessions. Additional resources were also an issue, such as obtaining medical supplies for self-management activities (i.e., blood pressure checks and glucometers strips); however, in several instances, nurses received the resources from health center directors or through working with participants. Lastly, there were issues with stakeholders feeling overwhelmed by the responsibilities of leading the intervention at their respective health centers, in addition to their existing commitments at the health center. While other health center staff did provide assistance with facilitation, in the future, scale-up efforts should conceptualize implementation strategies that support conducting sessions of local stakeholders.

In spite of the increased workload, there was still a high level of motivation in the implementation of the intervention. Stakeholders expressed determination to provide a high-quality session every week to participants, which in turn fostered better interactive activities and increased participation. The strong social relationships with participants provided an insight into commitment of stakeholders to conducting engaging sessions. Therefore, it became a cyclical process where strong foundational social relationships between participants and stakeholders resulted in higher-quality sessions and subsequently more engagement. Strong social relationships with participants should be emphasized as scale-up efforts continue in other health systems.

At the organizational level, motivation was related to a desire to achieve federal accreditation of the GAMs. Federal accreditation is dependent on several factors, such as number of members, programmatic structure, and biological and anthropometric measures recorded, provided by *MSD*, and contributed to report generation. The regional office was motivated to certify their GAMs to receive increased funding for groups delineated in the federal programmatic guidelines, therefore consistently communicating the urgency to hold weekly sessions and production of report to the leadership (10).

Consistent communication across ecological levels from participants, to facilitators and health centers directors, then to the regional health system office provided a continuous feedback and incorporation of recommendations during implementation. While the training for stakeholders and observational feedback provided were more straightforward approaches to building in learning, continuous cross-level communication facilitated through the implementation coordinator was a more flexible and equitable approach to integrating learning systems. Researchers also found these learning systems to be reflective of the interactive learning methods central to *MSD* (16). Furthermore, these lines of communications enacted a learning system where lessons learned could be rapidly implemented from week to week, and resources could be provided by the health center directors or regional office. The regional implementation coordinator role was central to this multilevel approach because he was able to facilitate a learning system rooted in dialogue among stakeholders after the initial training and during the entire scale-up process. This demonstrates effective methods for scaling chronic disease programs, which as seen in the literature have been difficult to accomplish (9, 33).

### Limitations

While the intervention was scaled to GAMs within Jurisdiction VI, there were limitations to this study. First, there was an initial dropout among participants in the beginning of implementation. This may have been due to the perception of participants that they would be receiving health services exclusively. Future efforts should better gauge commitment of participants to the 13-week intervention. Second, the limited number of participants in the study may limit the applicability to larger health systems. However, limited research resources did not allow for the research team to conduct the test in a larger health system. Lastly, the ecological approach utilized attempted to address several barriers; however, efforts were limited to being able to address every level equally such as competing policies to the MSD intervention.

## Conclusion

The multilevel approach provided a comprehensive framework on which to test a scalable unit, and as a result, a better understanding of the infrastructure and learning systems is necessary to scale-up. As evident from our approach, it is critical to receive buy-in and build cohesion from stakeholders across ecological levels. This facilitates strong lines of communication and resource sharing that ensure adherence to the intervention. While this implies breaking down traditional hierarchical power dynamics within health systems, it is necessary to foster a strong learning system—one based on feedback processes, collective problem-solving, and rapid solution-based implementation strategies. The approach also provided an insight into the need to assess adaptability of the intervention into diverse health center and system settings. Overall, this innovative approach to scaling *MSD* proved that a regional health system is a functional unit on which to base scale-up efforts and demonstrated an advantage to working outside of normative system dynamics when implementing and scaling interventions. Additionally, this study provides useful evidence for scaling a chronic disease intervention within an LMIC health system through a scientifically rigorous collaborative approach. Future directions should focus on further institutionalizing strategies to scaling chronic disease self-management programs, particularly within LMICs.

## Data Availability Statement

The raw data supporting the conclusions of this article will be made available by the authors, without undue reservation.

## Ethics Statement

The studies involving human participants were reviewed and approved by University of Arizona, Human Subject Protection Program. The patients/participants provided their written informed consent to participate in this study.

## Author Contributions

BA led the data collection, qualitative and quantitative analyses, writing of the manuscript, and assisted with stakeholder engagement. CR led to stakeholder engagement, designing approach of scale-up approach, and contributed to writing of the manuscript. MI and CD contributed to scale-up approach and writing of manuscript. JT assisted in stakeholder engagement, implementing the intervention, interpretation of results, and writing of manuscript. TN assisted in identifying funding for resources, statistical analysis, and writing of the manuscript. DG and PM assisted with writing the manuscript and interpretation of results. All authors have read and approved the manuscript.

## Conflict of Interest

The authors declare that the research was conducted in the absence of any commercial or financial relationships that could be construed as a potential conflict of interest.

## Publisher's Note

All claims expressed in this article are solely those of the authors and do not necessarily represent those of their affiliated organizations, or those of the publisher, the editors and the reviewers. Any product that may be evaluated in this article, or claim that may be made by its manufacturer, is not guaranteed or endorsed by the publisher.

## Abbreviations

BMI: Body mass index
CVD: Cardiovascular diseases
GAM: Grupos de Ayuda Mutua
HbA1c: Glycated hemoglobin
IF: Implementation fidelity
IHI: Institute for Healthcare Improvement
LMIC: Low- and middle-income country
MSD: Meta Salud Diabetes
SEM: Social–Ecological Model
SD: Standard deviation
SE: Standard error
T2DM: Type 2 diabetes mellitus.
